# Prevalence of Physical Activity and Sedentary Behaviors by Metropolitan Status in 4th-, 8th-, and 11th-Grade Students in Texas, 2004-2005

**Published:** 2008-12-15

**Authors:** Andrew E. Springer, Deanna M. Hoelscher, Steven H. Kelder, Brian Castrucci, Adriana Perez

**Affiliations:** Michael & Susan Dell Center for Advancement of Healthy Living, University of Texas School of Public Health; Michael & Susan Dell Center for Advancement of Healthy Living, University of Texas School of Public Health, Austin Regional Campus, Austin, Texas; Michael & Susan Dell Center for Advancement of Healthy Living, University of Texas School of Public Health, Austin Regional Campus, Austin, Texas; Texas Department of State Health Services; University of Texas School of Public Health, Brownsville Regional Campus, Brownsville, Texas

## Abstract

**Introduction:**

Research on geographic differences in children's physical activity (PA) engagement is limited. This study examined the prevalence of PA and sedentary behaviors in a probability sample of children in the 4th (mean age, 9.7 years; n = 7,907), 8th (mean age, 13.7 years; n = 8,827), and 11th (mean age 16.9 years; n = 6,456) grades by urban, suburban, and rural location in Texas.

**Methods:**

Using data from the 2004-2005 School Physical Activity and Nutrition (SPAN) study, we conducted logistic regression analyses stratified by sex to assess associations of 6 PA indicators and 2 sedentary behavior indicators with metropolitan status.

**Results:**

Urban 8th- and 11th-grade students reported the lowest prevalence of PA. Suburban or rural schools were significantly more likely than their urban counterparts to report higher school-based sports team participation in 8th graders (*P* = .001); higher vigorous PA (*P* = .01) and strengthening exercise (*P* = .01) in 11th-grade boys; and higher physical education attendance in 4th (*P* < .01) and 11th graders (*P* = .05). Sports team (*P* = .04) and other organized PA participation (*P* = .04) in urban 4th-grade girls and vigorous PA in urban 8th-grade boys (*P* = .04) were the only behaviors for which a significantly higher prevalence was reported compared with nonurban counterparts. We observed few significant geographic differences in prevalence of television watching and video game playing.

**Conclusion:**

Several significant differences in PA behaviors were found by metropolitan status in this sample of public school students in Texas. Research is needed on availability of PA opportunities and PA barriers by metropolitan status to better understand the lower prevalence estimates reported in older urban children.

## Introduction

Childhood obesity has been rising since the 1970s; recent estimates indicate that 17.1% of US children and adolescents are overweight (1). Obesity and its associated comorbidities are of particular concern for residents of rural areas in the United States, where obesity rates are higher in adults (2) and children (3-5). Children living in rural communities in the United States may be at heightened risk for obesity because of broader obstacles to health and well-being such high rates of poverty (6) and limited access to medical care (6,7).

Children's participation in regular physical activity (PA) is important for the prevention of obesity and is associated with several other health benefits, including the prevention of cardiovascular disease and type 2 diabetes, improved musculoskeletal health, and better mental and emotional health outcomes (8). Several large national health surveys in the United States have found leisure-time PA to be lower among adults who reside in rural areas compared with those in urban areas (2,9-11). In contrast to findings in adult studies, a study that used national data from the 2003 Youth Risk Behavior Survey found that urban adolescents generally report lower engagement in PA and higher engagement in sedentary behaviors than do their suburban and rural counterparts (12). Similarly, a 2008 study of 3,416 elementary school students in Iowa found urban children were the least active overall (5). Further research on urban and rural differences in PA in children is warranted to assess potential disparities in PA by metropolitan status.

The South is an important region to investigate urban and rural obesity prevention behaviors. Southern states have the largest proportion of rural children in the United States (5) and some of the highest prevalence rates of obesity. A national study of rural US counties found that counties in Louisiana, Mississippi, and Texas had the highest prevalence of obesity (13). Adolescent overweight has also been found to be higher in southern states (14). This study compares the prevalence of PA and sedentary behaviors among children who resided in urban, suburban, and rural metropolitan areas by using state data representative of public school students living in Texas. Recognizing that PA behaviors may vary by developmental age, this study examines PA and sedentary behaviors by metropolitan status in 4th-, 8th-, and 11th-grade students.

## Methods

### Study design

We used data from the 2004-2005 School Physical Activity and Nutrition (SPAN) study, which used a cross-sectional design to monitor the prevalence of child and adolescent overweight, diet, and PA behaviors of school-aged children in Texas (15). SPAN was designed to provide state representative data when stratified by ethnicity (African American, Hispanic, and white/other), sex, school grade (4th, 8th, and 11th), and Texas Health Service Region (15). The Committee for the Protection of Human Subjects at the University of Texas Health Science Center at Houston and the Texas Department of State Health Services institutional review board approved the study. Participating school districts reviewed study protocols for compliance with school district human subjects and research regulations. Further description of the SPAN study and methods can be found elsewhere (15).

### Participants

The SPAN study surveyed 3 grade levels of public school students with the aim of selecting distinct developmental ages of schoolchildren in elementary, middle, and high school. Data obtained from the Texas Education Agency (TEA) for public school enrollment during the 2003-2004 school year were used as the reference base for the sampling plan.

### Sampling

Texas is divided into 8 health service regions (HSRs) that serve as locally administered units by the Texas Department of State Health Services. The sampling plan for SPAN in 2004-2005 included collecting data representative of all HSRs. Using TEA criteria, each HSR was stratified into 3 school districts: urban center ("urban"), other urban/suburban ("suburban"), and rural. The largest school district in each HSR was designated as the "urban center" district. School districts from counties with populations of 25,000 to 650,000 were designated as "other urban/suburban," and districts from counties with populations fewer than 25,000 were designated as "rural." For the urban center district, 12 schools were randomly selected (4 for each grade level), with the selection probability proportional to the number of students. For other urban/suburban and rural schools, 5 districts were randomly chosen with selection probability proportional to the number of schools in the district. From each of these selected districts, 1 school from each level was randomly selected by using probability proportional sampling. For each selected school, at least 2 classes were sampled, for a total target of 50 students per school. Classes selected included courses that were required for all students at that grade level. The final sampling frame for the 2004-2005 survey contained 3,860 schools, which included 87% of all public school children in Texas. Further sampling details are available elsewhere (15).

The sample design incorporates a representative sample at the state level and by 3 major ethnic groups: African American, Hispanic, and white/other. For this study, students described their ethnicity from a list of 7 response options. "Other" ethnicity, which includes American Indian or Alaska Native, Asian, Native Hawaiian, and other Pacific Islander, was combined with white ethnicity because of small sample sizes for these other ethnic groups. No differences were found in body mass index or percentage overweight between the "other" subgroup and the white participants, thus providing a rationale for combining these groups. Further details are reported elsewhere (15).

### Measures

Six indicators of PA were examined: participation in vigorous physical activity (VPA) that made the respondent sweat and breathe hard for at least 20 minutes on 3 or more of the previous 7 days (based on adult recommendations for PA that were commonly used for children at the time of the study [16]); participation on at least 1 sports team in the previous year (for 8th and 11th grade, measures include participation on sports teams run by school and outside of school); current participation in other organized PA or lessons; participation in exercises to strengthen or tone muscles on 3 or more of the previous 7 days (8th and 11th grade only); and physical education (PE) class on 4 or more days during an average school week (Table 1). Two indicators assessed sedentary behavior (Table 2): watching television/video movies and playing computer/video games for 3 or more hours per day. These self-reported measures are identical to or adapted from the PA and media behavior measures used by the Centers for Disease Control and Prevention's 1999 Youth Risk Behavior Surveillance System (17), several of which are based on 7-day recall measures. Seven-day recall measures have adequate reliability and validity for 5th-, 8th-, and 11th-grade children in the United States (18). Questionnaires and protocols for SPAN were developed, pilot-tested, and assessed for reproducibility as part of the School-Based Nutrition Monitoring project (19,20). Among 4th-grade students, test-retest κ statistics ranged from 0.51 to 0.87 for PA and media behavior measures (19); in 8th-grade students, κ statistics ranged from 0.51 to 0.77 (20), indicating an acceptable-to-good level of reproducibility (Table 1). Standard protocols for administration of the survey are described elsewhere (19,20). Measures of sociodemographic characteristics included age, ethnicity, and percentage of economically disadvantaged students. Age was self-reported and was included as a continuous variable in the analysis. Ethnicity was also self-reported and is described earlier. Percentage of economically disadvantaged students was included as a measure of socioeconomic status (SES). This measure is based on TEA data from 2003-2004 and represents the percentage of economically disadvantaged students in the school. The TEA uses 3 categories to classify students as economically disadvantaged: eligible for free meals under the National School Lunch and Child Nutrition Program, eligible for reduced-cost meals under the National School Lunch and Child Nutrition Program, and other economically disadvantaged, which includes family with an annual income at or below the US poverty threshold.

### Data analysis

Sampling weights and adjustments are reported elsewhere (15). All estimates were weighted and statistical tests performed taking into account the sample design features. PA and sedentary behavior point prevalence estimates and confidence intervals were calculated by using Stata version 8 (StataCorp LP, College Station, Texas). Prevalence estimates were computed by grade level and metropolitan status (urban, suburban, and rural). Because PA patterns tend to differ for boys and girls (8), the analyses were also stratified by sex. For physical education class attendance, we also calculated the weighted mean of days attended in an average week. Simultaneous logistic regression analyses were conducted to assess the association between metropolitan status classifications and the PA and sedentary behavior outcomes, adjusting for age, ethnicity, and percentage of economically disadvantaged. All PA variables were dichotomized (present/absent) for the prevalence estimates and logistic regression analyses on the basis of the descriptions provided in the measures section. The score for percentage of economically disadvantaged students, obtained for all schools in the sample, was assigned to each student from a given school. This variable was treated as continuous in the analysis. Analyses without adjustment for percentage of economically disadvantaged students are not shown here. Overall, no major differences were found. Significance was established at *P* < .05.

## Results

The sex and age composition was similar across metropolitan status classifications for each grade (Table 3). Across grades, the proportion of African American students was highest in the urban stratum and lowest in the rural stratum, as expected from the sampling frame. Hispanic students were the largest ethnic group in the urban stratum, and white/other students were the largest ethnic group in the rural stratum across grades. The composition of Hispanic and white/other students was similar for the suburban stratum of 4th and 8th grades, and white/other students (50.3%) represented the largest ethnic group for the suburban stratum in 11th grade. The proportion of economically disadvantaged students was highest in urban students across grade levels, ranging from 59.9% of urban 11th-grade students to 69.7% of urban 4th-grade students (Table 3).

Prevalence estimates of engagement in PA and sedentary behavior by grade and metropolitan status were stratified by sex (Table 4 and Table 5). Urban students reported the largest decreases in PA from 4th to 11th grades, with significant decreases in PA in girls based on nonoverlapping confidence intervals found for sports team participation, vigorous PA, and participation in other PA/lessons. Suburban and rural boys' VPA increased from 4th to 11th grade, and urban boys' VPA decreased (Table 4).

With the exception of PE class attendance, PA prevalence estimates were similar across metropolitan status classifications for 4th-grade students. Prevalence of daily PE class attendance (≥4 days per week) in suburban and rural 4th-grade students was roughly double that of urban students (Table 5). Exploring mean PE days attended in an average week by metropolitan status, we found the mean daily PE attendance was lower for urban students in 4th (2.31; 95% confidence interval [CI], 2.09-2.54) and 11th grades (0.88; 95% CI, 0.12, 1.64) compared with same-grade suburban (4th-grade mean, 2.91; 95% CI, 2.62-3.19; 11th-grade mean, 2.17; 95% CI, 1.66-2.69) and rural students (4th-grade mean, 3.23; 95% CI, 2.84-3.62; 11th-grade mean, 1.78, 95% CI, 1.33-2.24).

For 8th- and 11th-grade students, the percentage of students who reported engaging in any of the 6 PA indicators examined was generally lower among urban students (Tables 4 and 5). Vigorous PA in 8th-grade urban boys was the only PA indicator that ranked higher compared with the suburban and rural status classifications. For all students, a higher percentage of 8th- and 11th-grade students reported playing on a sports team at school than on a sports team outside of school. This difference in sports team participation at school versus outside of school was highest in rural school students in 8th grade girls and boys (Table 4). Finally, generally similar prevalence estimates within each grade were found for sedentary behaviors of television watching and video game playing across the metropolitan status classifications (Table 6).

Differences in PA among metropolitan status classifications varied by PA indicator, grade level, and sex. Despite adjustments made for age, ethnicity, and percentage of economically disadvantaged students, suburban and rural students were more likely to report PA behavior than were urban students (Table 7 and Table 8). Suburban and rural 4th- and 11th-grade students were 2 to 3 times more likely than urban students to report attending PE on 4 or more days of the week (Table 8). In 8th grade, rural girls and boys were more than twice as likely to report participation on a sports team at school compared with urban counterparts from the same grade (Table 7). Suburban and rural 11th-grade boys were roughly twice as likely to report participating in vigorous PA on 3 or more days of the week compared with urban boys from the same grade level (Table 7), and 11th-grade suburban boys were 1.71 times more likely to report participation in strengthening exercise. No significant differences by metropolitan status were found for participation in other structured PA in 8th- and 11th-grade students (Table 8). Urban students generally reported lower prevalence of PA, but urban 4th-grade girls reported a higher likelihood of participating in sports teams and in other structured physical activities than did their nonurban counterparts (Tables 7 and 8), and urban 8th-grade boys were more likely to report engaging in vigorous PA than were their rural counterparts (Table 7). No significant differences among the metropolitan status classifications were found for 8th-grade students' participation in sports teams outside of school or engagement in strengthening exercise (Table 7).

Few significant differences were found by metropolitan status for sedentary behaviors, and the direction of the significant associations was mixed (Table 9). In 4th grade, suburban and rural girls were 1.54 times more likely to report watching at least 3 hours of television per day compared with their urban counterparts. In 8th grade, rural boys were more than twice as likely to play computer/video games compared to urban boys of the same age. In 11th grade, suburban boys were less likely to watch at least 3 hours of television per day compared with urban boys. No significant differences in television watching by metropolitan status were found for 8th grade boys, and no significant differences in computer/video game playing were found for 4th- or 11th-grade girls and boys (Table 9).

## Discussion

We aimed to assess potential disparities in child and adolescent PA behaviors by residence in urban, suburban, or rural areas. Contrary to findings of lower prevalence of PA among adults in rural areas (2,9-11), we found that urban 8th- and 11th-grade students generally reported lower prevalence of PA and urban 4th-grade students reported lower daily PE participation than did their rural or suburban counterparts. This finding is supported in part by previous research of elementary (5) and high school students (12) that found urban children reported the lowest prevalence of PA behaviors.

The lower prevalence of PA in 8th- and 11th-grade urban students may result from greater concerns about the safety of children who live in urban environments. Several studies have documented an inverse association between parent and child perceptions of neighborhood safety and children's engagement in PA (21-23). Urban residents may have more concerns about neighborhood safety because violent and property crime rates are higher in urban than in suburban and rural areas (24). A national report of school crime and safety in the United States found that urban students were more likely than suburban and rural students to report fear of attack at school, on the way to or from school, and away from school (25). Parental anxiety about neighborhood safety is negatively correlated with children's PA levels, and urban parents express more anxiety about neighborhood safety than do suburban parents (22).

Our finding of a higher prevalence of sports team participation and other structured PA participation in 4th-grade students and lower prevalence of these PA behaviors in 11th-grade students across metropolitan status corroborates previous research findings that engagement in PA declines as children mature through adolescence (8). We found that between 4th and 11th grade, urban students reported the largest decline in sports team participation and vigorous PA, and among the largest declines in other structured PA participation. A longitudinal study of adolescent PA found the decline in the total amount of PA during adolescence to be primarily a function of a decrease in the number of activities rather than a decrease in the time spent on specific activities (26). Our findings of a lower prevalence of participation in team sports and other organized PA among urban 8th- and 11th-grade students suggest that older children in urban environments may face greater barriers to participation in structured PA. Barriers that merit further investigation to understand lower PA in urban students include the possibility of greater competition for participation on sports teams, higher costs for sports and other PA participation, and decreasing availability of structured PA opportunities for the higher grade levels.

Given that our results indicated higher prevalence of participation in school-based sports compared with sports by outside organizations across metropolitan status classifications, schools appear to be an important context for providing sports participation opportunities across metropolitan status. Our finding that rural 8th-grade boys and girls are more than twice as likely as their urban counterparts to participate in school sports may indicate that more opportunities for school-based sports are available in rural areas than in urban areas. A stronger social norm for sports team participation among rural students may also be a factor. Further research is needed to assess the availability of and access to organized school- and community-based physical activity opportunities by metropolitan status to better understand our finding of less participation in organized PA among urban 8th- and 11th-grade students.

The lower prevalence of some PA behaviors in urban students may also result from less access to recreational areas and facilities. Closer proximity and higher density of exercise facilities have been associated with increased frequency of exercise (27). In a study of commercial PA-related outlets across the United States, suburban areas were significantly more likely than urban and rural areas to have physical fitness facilities, membership sports and recreation clubs, dance studios, schools and halls, and public golf courses (28). Lower access to areas of recreation such as parks is another factor that may account for lower prevalence of PA behaviors in urban students observed in this study. Access to parks has been found to be associated with higher engagement in PA behaviors in adolescent girls (29). A study based on the 2003 California Health Interview Survey found that teens living in neighborhoods with a greater concentration of crowded households and a higher proportion of residents living in poverty had less access to parks and were less physically active (30). Research is needed to assess differences in access to recreational facilities by metropolitan status as a potential explanation for lower PA in urban students.

Our data indicate that urban 4th- and 11th-grade students reported significantly less PE attendance than their nonurban counterparts, with a mean difference of approximately 1 day per week compared with rural students. A study of 277 elementary schools in Montreal found that suburban location was associated with greater opportunity for student PA in schools (31). In a national study of PA in US high school students (12), point prevalence estimates of PE attendance on at least 1 day per week were the lowest for urban students. With the passage of Texas Senate Bill 530, elementary and middle schools in Texas are now required to implement 30 minutes of PA per day or 125 minutes of PA per week. As such, this requirement may reduce the disparity in PE minutes by metropolitan status over time, provided that schools consider PE an opportunity for addressing PA-mandated minutes. In high schools, however, where no recent PA legislation has been passed in Texas, this disparity in PE merits further attention to identify factors such as local district policies, competing academic interests, and budget constraints that may account for the lower PE participation in urban students.

Sedentary behaviors such as television watching have been associated with child overweight (32) and have been hypothesized to displace time spent in PA (8). Our prevalence estimates of television watching for at least 3 hours per day in this sample of 11th-grade students from Texas are similar to national estimates for US high school students (33). In observing sedentary behavior by metropolitan status, we found few significant differences among the grade levels examined. The similar prevalence estimates of sedentary behavior in urban, suburban, and rural students is supported in part by our previous study of US high school students in which we reported no significant differences by metropolitan status in television watching for either male or female students or for computer/video game playing in male students (12). In contrast to these findings, adolescents living in inner city neighborhoods of low SES and urban locations with diverse race/ethnicity were significantly more likely to report at least 14 hours of television/video viewing and video/computer gaming per week than those living in newer suburban developments (34). These results are most likely due to differences in the way in which geographic location was defined; Nelson et al used a cluster analysis approach to identify specific, nonoverlapping patterns of neighborhoods that depart from the traditional classification of urban, rural, and suburban (34). We recognize the potential for better differentiation of location based on an approach that takes into consideration other neighborhood characteristics, but our findings nonetheless suggest that a large proportion of 4th-, 8th-, and 11th-grade students across metropolitan status classifications (range, 28.0%-62.2%) engage in long hours of media-related sedentary behavior every day.

### Limitation*s*

The findings in this study are subject to 3 limitations. First, self-reported PA may be overestimated (35). Whether this bias operates differently among adolescents from different metropolitan status designations is unclear. Recognizing this limitation, we found evidence of reliability and validity for self-reported PA and sports team participation in children (35). Second, although we attempted to control for the effects of SES by adjusting for school composition of economically disadvantaged students, we did not have individual-level data on SES for our study sample. Because access to PA facilities and recreational areas varies with SES (28,36), we cannot completely rule out SES as an explanation for PA differences by metropolitan status. A third limitation relates to the challenges inherent in the definition of metropolitan status. Recently, prevalence estimates of teenage smoking and drinking were found to vary based on 4 definitions of metropolitan status or "locational" variation (37). Future research in this area may benefit from innovative approaches for defining location such as those used by Nelson et al (34), which take into account factors such as street connectivity, crime and safety, income, and access to PA facilities. These approaches may allow for greater specification of neighborhood type and identification of specific neighborhood characteristics associated with overweight and PA.

### Conclusions

Our findings of a low prevalence of PA behaviors in 11th-graders compared with 4th-graders and a high prevalence of long hours of daily television watching and computer/video game playing across grades and metropolitan status classifications underscore the continued need to promote physically active lifestyles in Texas children, regardless of where in the state they live. At the same time, the generally lower prevalence of reported PA behaviors for urban 8th- and 11th-grade students and PE attendance in urban 4th- and 11th-grade students suggests the need to heighten PA promotion efforts and opportunities for urban children. Given the limited research on urban and rural differences in children's PA, further research is warranted to confirm our findings of a generally lower prevalence of PA in urban students. Research on the availability of school, neighborhood, and larger community PA opportunities and potential barriers to participation by urban, suburban, and rural location would provide further insights into the geographic factors that may affect PA and sedentary behavior engagement in children.

## Figures and Tables

**Table 1:** Self-Reported Physical Activity Measures for 4th-, 8th-, and 11th-Grade Students, School Physical Activity and Nutrition Study (SPAN) Questionnaire, Texas, 2004-2005

4th-Grade Students	κ^a^,^b^	8th- and 11th-Grade Students	κ^b^,^c^
**Vigorous physical activity**		**Vigorous physical activity**	
Yesterday, did you do any exercise that made your heart beat fast and made you breathe hard for at least 20 minutes? (For example: basketball, soccer, running or jogging, fast dancing, swimming laps, tennis, fast bicycling, or similar aerobic activities.) (Response options: yes, no)	0.71	On how many of the past 7 days did you exercise or take part in physical activity that made your heart beat fast and made you breathe hard for at least 20 minutes? (For example: basketball, soccer, running or jogging, fast dancing, swimming laps, tennis, fast bicycling, or similar aerobic activities). (Response options: 0 d, 1 d, 2 d, 3 d, 4 d, 5 d, 6 d, 7 d)	0.77
**Sports team participation**		**Sports team participation**	
During the past 12 months, on how many sports teams did you play? Sports teams include soccer, basketball, softball, swimming, gymnastics, cheerleading, wrestling, track, football, dance, tennis, and volleyball teams. (Response options: 0 teams, 1 team, 2 teams, 3 or more teams)	0.51	During the past 12 months, on how many sports teams run by your school did you play (do not include PE classes)? Sports teams include soccer, basketball, baseball, swimming, gymnastics, wrestling, track, football, tennis, and volleyball teams. (Response options: same as 4th grade)	0.71
		During the past 12 months, on how many sports teams run by organizations outside of your school (like the park district, summer leagues) did you play? Sports teams include soccer, basketball, baseball, swimming, gymnastics, wrestling, track, football, tennis, and volleyball teams. (Response options: same as above)	0.60
		**Participation in exercises to strengthen or tone muscles**	
		On how many of the past 7 days did you do exercises to strengthen or tone your muscles, such as push-ups, sit-ups, or weight lifting? (Response options: 0 d, 1 d, 2 d, 3 d, 4 d, 5 d, 6 d, 7 d)	0.71
**Attendance in PE class**		**Attendance in PE class**	
Last week, on how many days did you go to physical education (PE) or gym classes? (Response options: 0 d, 1 d, 2 d, 3 d, 4 d, 5 d)	0.87	In an average week when you are in school, on how many days do you go to PE classes? (Response options: same as 4th grade)	0.71

Abbreviation: PE, physical education.

a Penkilo et al (19).

b κ statistics based on a test-retest administration of questionnaire on the same day of the survey, with a time interval ≥2 hours.

c Hoelscher et al (20).

**Table 2:** Self-Reported Sedentary Behavior Measures for 4th-, 8th-, and 11th-Grade Students, School Physical Activity and Nutrition Study (SPAN) Questionnaire, Texas, 2004-2005

4th-Grade Students	κ^a^,^b^	8th- and 11th-Grade Students	κ^b^,^c^
**Watching television/video movies**		**Watching television/video movies**	
Yesterday, how many hours did you watch TV or video movies away from school? (Responses options: I didn't watch TV yesterday, 1 h, 2 h, 3 h, 4 h, 5 h, 6 h or more)	0.82	How many hours per day do you usually watch TV or video movies away from school? (Response options: I don't watch TV or video movies, 1 h, 2 h, 3 h, 4 h, 5 h, 6 h or more)	0.51
**Computer/video game playing**		**Computer/video game playing**	
How many hours per day do you usually play video games like Nintendo, Sega, Playstation, Xbox, GameBoy or arcade games away from school? (Response options: I don't play video games, 1 h, 2 h, 3 h, 4 h, 5 h, 6 h or more)	0.83	(Same as 4th grade)	0.71

a Penkilo et al (19).

b κ statistics based on a test-retest administration of questionnaire on the same day of the survey, with a time interval of ≥2 hours.

c Hoelscher et al (20).

**Table 3:** Demographic Characteristics of 4th-, 8th-, and 11th-Grade Students by Metropolitan Status, School Physical Activity and Nutrition (SPAN) Questionnaire, Texas, 2004-2005^a^

Characteristic	Metropolitan Status^b^		

	Urban	Suburban	Rural
**4th-grade students (n = 7,907)**			
**Sex**			
Female	48.9 (44.7-53.1)	48.8 (44.9-52.8)	49.1 (46.2-52.0)
Male	51.1 (46.9-55.3)	51.2 (47.2-55.1)	50.9 (47.9-53.8)
**Age, mean (95% CI), y**	9.7 (9.7-9.8)	9.7 (9.6-9.8)	9.8 (9.7-9.9)
**Race/ethnicity**			
African American	16.6 (9.2-23.9)	13.3 (8.7-17.9)	9.6 (5.3-13.8)
Hispanic	62.4 (48.9-75.9)	43.1 (35.6-50.7)	38.2 (33.9-42.4)
White/other^c^	21.0 (6.4-35.6)	43.6 (36.5-50.7)	52.2 (47.3-57.3)
**School composition**			
Economic disadvantage^d^	69.7 (67.9-71.4)	64.4 (63.7-65.2)	59.5 (58.5-60.5)
**8th-grade students (n = 8,827)**			
**Sex**			
Female	49.6 (42.9-56.3)	49.2 (42.9-56.3)	48.6 (40.7-56.4)
Male	50.4 (43.7-57.1)	50.8 (48.1-53.5)	51.4 (43.6-59.3)
**Age, mean (95% CI), y**	13.7 (13.6-13.8)	13.7 (13.6-13.8)	13.8 (13.7-13.9)
**Race/ethnicity**			
African American	19.1 (12.0-26.2)	14.4 (7.3-21.5)	10.1 (5.3-14.9)
Hispanic	56.0 (47.6-64.5)	40.4 (30.3-50.4)	30.5 (21.4-39.7)
White/other^c^	24.9 (15.1-34.7)	45.2 (34.8-55.7)	59.3 (48.3-70.4)
**School composition**			
Economic disadvantage^d^	74.3 (73.0-75.5)	54.3 (53.7-55.0)	55.3 (54.2-56.4)
**11th-grade students (n = 6,456)**			
**Sex**			
Female	52.9 (47.3-58.5)	49.2 (43.8-54.6)	49.1 (46.6-51.7)
Male	47.1 (41.5-52.7)	50.8 (45.4-56.2)	51.4 (48.3-53.4)
**Age, mean (95% CI), y**	16.9 (16.7-17.0)	16.7 (16.7-16.8)	16.8 (16.8-16.9)
**Race/ethnicity**			
African American	22.1 (11.3-32.8)	13.6 (7.3-19.9)	9.2 (4.7-13.8)
Hispanic	49.3 (41.2-58.9)	36.1 (30.2-42.0)	26.9 (14.0-39.8)
White/other^c^	28.6 (14.6-42.7)	50.3 (41.7-58.9)	63.9 (49.8-78.0)
**School composition**			
Economic disadvantage^d^	59.9 (58.3-61.6)	37.6 (36.8-38.3)	41.8 (40.5-43.0)

a Results based on weighted analysis.

b Data are presented as % (95% confidence interval) unless otherwise indicated.

c "Other" includes the following races: American Indian or Alaska Native, Asian, and Native Hawaiian or other Pacific Islander.

d Data obtained from Texas Education Agency. Defined as mean percentage of students in a school that are classified as economically disadvantaged. Data presented represent mean percentage and 95% CI.

**Table 4:** Prevalence of Vigorous Physical Activity and Sports Team Participation by Metropolitan Status Among 4th-, 8th-, and 11th-Grade Students, School Physical Activity and Nutrition (SPAN) Questionnaire, Texas, 2004-2005^a^

Grade	Vigorous PA^b^		Played on ≥1 SportsTeam at School^c^		Played on ≥1 SportsTeam Outside School^c^		Played on ≥1 SportsTeam^c ^ (Run by School orOutside Organization)	

	Girls	Boys	Girls	Boys	Girls	Boys	Girls	Boys
**4th-grade students**								
Urban	68.6 (62.6-74.6)	66.5 (59.3-73.8)	NA	NA	NA	NA	73.4 (67.8-79.0)	72.5 (62.5-82.5)
Suburban	68.0 (63.2-72.9)	68.5 (64.0-73.0)	NA	NA	NA	NA	66.8 (61.8-71.8)	76.4 (70.7-82.1)
Rural	72.6 (67.2-77.9)	66.8 (62.6-70.9)	NA	NA	NA	NA	72.8 (66.6-78.9)	79.7 (76.8-82.6)
**8th-grade students**								
Urban	71.6 (58.8-84.4)	85.6 (79.2-92.0)	41.0 (35.0-47.1)	55.5 (36.8-74.2)	40.7 (22.3-59.0)	46.6 (27.4-65.8)	58.8 (46.4-71.2)	68.2 (50.5-85.9)
Suburban	74.4 (71.0-77.8)	81.4 (75.4-87.3)	49.3 (41.5-57.1)	61.7 (57.1-66.3)	41.2 (36.6-45.8)	50.1 (42.6-57.6)	64.8 (59.9-69.7)	75.4 (69.2-81.6)
Rural	75.9 (71.1-80.7)	82.7 (78.2-87.2)	65.1 (56.7-73.6)	73.0 (65.2-80.8)	49.2 (46.3-52.0)	51.8 (47.1-56.5)	75.0 (70.5-79.4)	80.4 (73.8-87.0)
**11th-grade students**								
Urban	42.2 (27.1-57.5)	60.0 (51.2-68.8)	34.4 (25.5-43.4)	31.4 (6.8-56.1)	16.9 (1.0-32.8)	22.9 (4.4-41.3)	39.8 (26.7-52.8)	37.6 (7.9-67.2)
Suburban	60.5 (49.7-71.3)	75.1 (69.8-80.3)	48.8 (36.7-61.0)	54.7 (47.2-62.3)	32.0 (26.2-37.8)	32.7 (26.8-38.7)	56.2 (46.3-66.2)	61.1 (53.1-69.1)
Rural	50.0 (42.8-57.2)	75.8 (70.4-81.2)	41.9 (38.3-45.6)	51.6 (46.5-56.7)	30.2 (23.7-36.6)	36.5 (29.9-43.2)	52.9 (49.3-56.5)	59.6 (50.7-68.4)

Abbreviations: PA, physical activity; NA, not available.

a Results based on weighted analysis. Data are presented as % (95% confidence interval).

b Exercised or participated in physical activities that made them sweat and breathe hard for ≥20 minutes on ≥3 of the previous 7 days.

c Played on ≥1 sports team during past 12 months.

**Table 5:** Organized Physical Activity Participation, Strengthening Exercise, and Physical Education Attendance by Metropolitan Status Among 4th-, 8th-, and 11th-Grade Students, School Physical Activity and Nutrition (SPAN) Questionnaire, Texas, 2004-2005^a^

Grade	Other Organized PA or Lessons		Strengthening Exercise^b^		PE Attendance ≥4 d/week^c^	

	Girls	Boys	Girls	Boys	Girls	Boys
**4th-grade students**						
Urban	58.5 (52.7-64.4)	36.8 (24.5-49.0)	NA	NA	22.3 (16.3-28.4)	22.2 (16.1-28.3)
Suburban	49.8 (43.2-56.4)	34.9 (28.8-40.9)	NA	NA	39.9 (28.8-51.0)	40.7 (27.5-53.8)
Rural	50.3 (46.0-54.6)	32.4 (25.5-39.2)	NA	NA	46.7 (31.4-61.9)	47.1 (33.6-60.6)
**8th-grade students**						
Urban	31.9 (26.4-37.5)	25.9 (17.7-34.0)	38.4 (31.9-44.8)	61.7 (37.2-86.3)	49.0 (10.7-87.4)	56.8 (16.5-97.0)
Suburban	35.5 (30.7-40.4)	27.4 (23.0-31.9)	46.1 (40.2-52.1)	63.7 (57.4-69.9)	42.4 (33.9-51.0)	55.2 (48.1-62.2)
Rural	37.1 (29.6-44.6)	20.9 (16.6-25.2)	47.1 (40.6-53.6)	72.6 (64.8-80.3)	68.1 (49.2-86.9)	60.1 (44.8-75.4)
**11th-grade students**						
Urban	26.4 (20.1-32.7)	12.6 (2.6-22.6)	51.5 (30.1-72.9)	48.9 (44.9-53.0)	11.0 (−.01-22.0)	15.0 (1.9-28.1)
Suburban	33.4 (23.7-43.2)	19.1 (15.7-22.6)	39.8 (31.2-48.4)	61.2 (52.2-70.2)	37.5 (20.8-54.1)	39.0 (30.2-47.7)
Rural	25.0 (22.6-27.4)	20.6 (14.6-26.6)	29.5 (17.9-41.1)	56.6 (45.5-67.8)	27.3 (14.9-39.7)	39.0 (28.5-49.5)

Abbreviations: PA, physical activity; PE, physical education; NA, not available.

a Results based on weighted analysis. Data are presented as % (95% confidence interval).

b Did exercises to strengthen or tone muscles on ≥3 of the past 7 days.

c Attended physical education class on ≥4 days during an average school week.

**Table 6:** Sedentary Behaviors Among 4th-, 8th-, and 11th-Grade Students, by Metropolitan Status, School Physical Activity and Nutrition (SPAN) Questionnaire, Texas, 2004-2005^a^

Grade	Watched Television for ≥3 h/day		Played Computer/Video Games for ≥3 h/day	

	Girls	Boys	Girls	Boys
**4th-grade students**				
Urban	26.9 (21.2-32.5)	36.6 (27.9-45.3)	8.0 (1.6-14.5)	30.0 (25.6-34.4)
Suburban	29.1 (26.1-32.1)	35.5 (31.2-39.8)	9.5 (7.1-12.0)	29.6 (24.7-34.5)
Rural	24.9 (20.2-29.7)	30.8 (25.9-35.7)	8.3 (4.5-12.1)	32.6 (27.5-37.8)
**8th-grade students**				
Urban	60.8 (57.0-64.7)	50.3 (42.9-57.7)	3.3 (1.0-5.9)	31.4 (24.1-38.7)
Suburban	50.9 (44.3-57.5)	49.2 (36.9-61.4)	4.8 (3.3-6.3)	24.8 (17.9-31.8)
Rural	51.4 (46.5-56.4)	54.1 (45.2-63.0)	3.6 (2.4-4.8)	41.9 (35.2-48.5)
**11th-grade students**				
Urban	40.4 (25.9-54.9)	53.5 (48.3-58.7)	0.9 (−0.3-2.1)	9.7 (1.1-18.4)
Suburban	39.4 (33.9-44.8)	45.2 (38.6-51.8)	0.8 (0.3-1.4)	17.1 (12.2-22.0)
Rural	40.4 (34.2-46.5)	45.9 (39.3-52.5)	1.2 (0.6-1.8)	14.5 (10.6-18.4)

a Results based on weighted analysis. Data are presented as % (95% confidence interval).

**Table 7:** Participation in Vigorous Physical Activity and Sports Teams Among 4th-, 8th-, and 11th-Grade Students, by Metropolitan Status, School Physical Activity and Nutrition (SPAN) Questionnaire, Texas, 2004-2005^a^

Grade	Vigorous PA ≥3 d/week^b^				Played on ≥1 SportsTeam at School^c^				Played on ≥1 Sports Team Outside of School^c^				Played on ≥1 Sports Teams^c^ (Run by School or Other Organization)			

	Girls, AOR (95% CI)	*p* Value	Boys, AOR (95% CI)	*P* Value	Girls, AOR (95% CI)	*P* Value	Boys, AOR (95% CI)	*P* Value	Girls, AOR (95% CI)	*P* Value	Boys, AOR (95% CI)	*P* Value	Girls, AOR (95% CI)	*P* Value	Boys, AOR (95% CI)	*P* Value
**4th-grade students**																
Urban	1.00		1.00		NA		NA		NA		NA		1.00		1.00	
Suburban	0.95 (0.64-1.43)	.82	1.04 (0.67-1.62)	.86	NA	NA	NA	NA	NA	NA	NA	NA	0.61 (0.43-0.87)	.007	1.12 (0.55-2.26)	.76
Rural	1.18 (0.75-1.86)	.47	0.84 (0.53-1.31)	.43	NA	NA	NA	NA	NA	NA	NA	NA	0.77 (0.47-1.24)	.27	1.44 (0.70-2.95)	.31
**8th-grade students**																
Urban	1.00		1.00		1.00		1.00		1.00		1.00		1.00		1.00	
Suburban	0.88 (0.53-1.46)	.62	0.53 (0.28-1.02)	.06	1.20 (0.74-1.94)	.46	1.25 (0.67-2.34)	.47	0.79 (0.48-1.31)	.36	0.95 (0.42-2.17)	.90	1.01 (0.64-1.60)	.97	1.16 (0.45-3.02)	.75
Rural	0.81 (0.46-1.41)	.45	0.51 (0.27-0.96)	.04	2.13 (1.11-4.10)	.02	2.10 (1.01-4.37)	.04	0.96 (0.54-1.69)	.88	0.94 (0.40-2.18)	.88	1.44 (0.81-2.54)	.21	1.44 (0.47-4.36)	.52
**11th-grade students**																
Urban	1.00		1.00		1.00		1.00		1.00		1.00		1.00		1.00	
Suburban	1.84 (0.81-4.18)	.14	1.97 (1.14-3.41)	.02	1.93 (0.93-3.99)	.08	2.63 (0.82-8.44)	.10	2.19 (0.73-6.55)	.16	1.62 (0.63-4.13)	.31	2.01 (0.92-4.40)	.08	2.63 (0.75-9.17)	.13
Rural	1.25 (0.57-2.76)	.58	1.96 (1.12-3.44)	.02	1.50 (0.82-2.72)	.18	2.30 (0.70-7.58)	.17	2.10 (0.69-6.40)	.19	2.07 (0.77-5.54)	.15	1.81 (0.87-3.74)	.11	2.48 (0.67-9.26)	.17

Abbreviations: AOR, adjusted odds ratio; PA, physical activity; CI, 95% confidence interval; NA, not applicable.

a Results based on a weighted, simultaneous regression analysis adjusted for age, ethnicity, and school proportion of economically disadvantaged students.

b Exercised or participated in physical activities that made them sweat and breathe hard for ≥20 minutes on ≥3 of the previous 7 days.

c Played on ≥1 sports teams during previous 12 months.

**Table 8:** Adjusted Odds Ratios for Participation in Other Organized Physical Activity, Strengthening Exercise, and Physical Education Class by Metropolitan Status Among 4th-, 8th-, and 11th-Grade Students, School Physical Activity and Nutrition (SPAN) Questionnaire, Texas, 2004-2005^a^

Grade	Other Organized PA or Lessons				Strengthening Exercise^b^				Attended PE on ≥4 days^c^			

	Girls, AOR (95% CI)	*P* Value	Boys, AOR (95% CI)	*P* Value	Girls, AOR (95% CI)	*P* Value	Boys, AOR (95% CI)	*P* Value	Girls, AOR (95% CI)	*P* Value	Boys, AOR (95% CI)	*P* Value
**4th-grade students**												
Urban	1.00		1.00		NA	NA	NA	NA	1.00		1.00	
Suburban	0.68 (0.46-1.00)	.049	0.96 (0.51-1.81)	.89	NA	NA	NA	NA	2.30 (1.25-4.22)	.007	2.75 (1.43-5.27)	.003
Rural	0.68 (0.47-0.97)	.03	0.88 (0.45-1.69)	.69	NA	NA	NA	NA	3.06 (1.36-6.86)	.007	3.75 (1.78-7.88)	.001
**8th-grade students**												
Urban	1.00		1.00		1.00		1.00		1.00		1.00	
Suburban	1.00 (0.71-1.42)	.98	1.14 (0.71-1.80)	.59	1.17 (0.77-1.76)	.46	0.91 (0.37-2.22)	.84	0.53 (0.14-1.96)	.34	0.64 (0.17-2.41)	.50
Rural	0.98 (0.57-1.68)	.93	0.79 (0.49-1.29)	.35	1.07 (0.65-1.79)	.78	1.30 (0.53-3.22)	.56	1.24 (0.31-4.89)	.76	0.69 (0.18-2.61)	.58
**11th-grade students**												
Urban	1.00		1.00		1.00		1.00		1.00		1.00	
Suburban	1.61 (0.81-3.21)	.17	1.61 (0.61-4.23)	.39	0.57 (0.20-1.58)	.27	1.71 (1.12-2.60)	.01	4.45 (1.16-17.04)	.03	3.60 (1.29-10.07)	.02
Rural	1.03 (0.58-1.81)	.93	1.78 (0.64-4.92)	.26	0.36 (0.11-1.20)	.09	1.27 (0.69-2.33)	.45	2.82 (0.75-1.59)	.12	3.72 (1.26-10.97)	.02

Abbreviations: PA, physical activity; PE, physical education; AOR, adjusted odds ratio; CI, 95% confidence interval; NA, not available.

a Results based on a weighted, simultaneous regression analysis adjusted for age, ethnicity, and school composition of economically disadvantaged students.

b Did exercises to strengthen or tone muscles on ≥3 of the previous 7 days.

c Attended PE class on ≥4 days during an average school week.

**Table 9:** Participation in Sedentary-Related Behaviors by Metropolitan Status Among 4th-, 8th-, and 11th-Grade Students, School Physical Activity and Nutrition (SPAN) Questionnaire, Texas, 2004-2005^a^

Grade	Watched Television/Video Movies for ≥3 h/day				Played Computer/Video Games for ≥3 h/day			

	Girls, AOR (95% CI)	*P* Value	Boys, AOR (95% CI)	*P* Value	Girls, AOR (95% CI)	*P* Value	Boys, AOR (95% CI)	*P* Value
**4th-grade students**								
Urban	1.00		1.00		1.00		1.00	
Suburban	1.54 (1.18-2.02)	.002	1.09 (0.75-1.58)	.66	1.28 (0.55-3.01)	.56	1.06 (0.70-1.60)	.77
Rural	1.40 (0.99-1.98)	.05	0.89 (0.59-1.33)	.56	1.17 (0.44-3.08)	.75	1.32 (0.91-1.91)	.15
**8th-grade students**								
Urban	1.00		1.00		1.00		1.00	
Suburban	0.90 (0.67-1.22)	.50	1.31 (0.99-1.74)	.06	1.68 (0.77-3.65)	.19	0.89 (0.67-1.19)	.44
Rural	1.13 (0.80-1.60)	.49	1.93 (0.99-3.72)	.05	1.37 (0.58-3.25)	.47	2.27 (1.37-3.76)	.002
**11th-grade students**								
Urban	1.00		1.00		1.00		1.00	
Suburban	1.30 (0.60-2.85)	.50	0.72 (0.53-0.97)	.03	1.32 (0.29-5.95)	.72	1.94 (0.74-5.13)	.18
Rural	1.53 (0.68-3.42)	.30	0.78 (0.56-1.09)	.15	2.55 (0.62-10.53)	.19	1.64 (0.62-4.36)	.31

Abbreviations: OR, odds ratio; CI, 95% confidence interval.

a Results based on a weighted, simultaneous regression analysis adjusted for age, ethnicity, and school proportion of economically disadvantaged students.
